# Silicon isotope constraints on terrestrial planet accretion

**DOI:** 10.1038/s41586-023-06135-z

**Published:** 2023-06-14

**Authors:** Isaac J. Onyett, Martin Schiller, Georgy V. Makhatadze, Zhengbin Deng, Anders Johansen, Martin Bizzarro

**Affiliations:** 1 Centre for Star and Planet Formation, Globe Institute, University of Copenhagen, Copenhagen, Denmark; 2 Lund Observatory, Department of Astronomy and Theoretical Physics, Lund University, Lund, Sweden; 3 Institut de Physique du Globe de Paris, Université de Paris Cité, Paris, France

**Keywords:** Geochemistry, Geochemistry, Inner planets

## Abstract

Understanding the nature and origin of the precursor material to terrestrial planets is key to deciphering the mechanisms and timescales of planet formation^1^. Nucleosynthetic variability among rocky Solar System bodies can trace the composition of planetary building blocks^2–5^. Here we report the nucleosynthetic composition of silicon (μ^30^Si), the most abundant refractory planet-building element, in primitive and differentiated meteorites to identify terrestrial planet precursors. Inner Solar System differentiated bodies, including Mars, record μ^30^Si deficits of −11.0 ± 3.2 parts per million to −5.8 ± 3.0 parts per million whereas non-carbonaceous and carbonaceous chondrites show μ^30^Si excesses from 7.4 ± 4.3 parts per million to 32.8 ± 2.0 parts per million relative to Earth. This establishes that chondritic bodies are not planetary building blocks. Rather, material akin to early-formed differentiated asteroids must represent a major planetary constituent. The μ^30^Si values of asteroidal bodies correlate with their accretion ages, reflecting progressive admixing of a μ^30^Si-rich outer Solar System material to an initially μ^30^Si-poor inner disk. Mars’ formation before chondrite parent bodies is necessary to avoid incorporation of μ^30^Si-rich material. In contrast, Earth’s μ^30^Si composition necessitates admixing of 26 ± 9 per cent of μ^30^Si-rich outer Solar System material to its precursors. The μ^30^Si compositions of Mars and proto-Earth are consistent with their rapid formation by collisional growth and pebble accretion less than three million years after Solar System formation. Finally, Earth’s nucleosynthetic composition for *s*-process sensitive (molybdenum and zirconium) and siderophile (nickel) tracers are consistent with pebble accretion when volatility-driven processes during accretion and the Moon-forming impact are carefully evaluated.

## Abstract (web summary)

The nucleosynthetic composition of silicon in meteorites indicates that material akin to early-formed differentiated asteroids must represent a major constituent of terrestrial planets such as Earth and Mars.

## Main

The classical theory for terrestrial planet formation involves a phase of giant impacts between embryos over timescales of 50–100 Myr (ref. ^6^). This long-standing paradigm has recently been challenged by astrophysical observations^7^ and isotopic evidence for rapid planetary accretion^8^. Although the hafnium–tungsten age of Earth has been used to argue for its protracted accretion >30 Myr after Solar System formation, the tungsten isotope composition of Earth’s mantle is consistent with rapid accretion of proto-Earth provided that the Moon-forming giant impact occurred late^9^. Thus, the mechanisms of terrestrial planet formation are still debated and theories such as pebble accretion allowing rapid formation timescales have emerged^10^. In this model, streaming instabilities facilitate the rapid formation of 100-km-sized bodies that grow to form the terrestrial planets by the accretion of millimetre-sized pebbles within the 3–5-Myr protoplanetary disk lifetime^11^.

The Solar System’s nucleosynthetic isotopic variability can provide insights into the nature of the material precursor to terrestrial planets and, hence, their formation pathways. However, care must be taken in interpreting nucleosynthetic data as some tracers record only a minor fraction of a body’s accretion history. Highly siderophile elements such as molybdenum (Mo) and ruthenium (Ru) essentially reside in planetary cores such that their depleted abundance in the silicate portion of rocky bodies documents only the final stage of a planet’s accretion^5^. Moreover, siderophile elements are strongly affected by metal–silicate equilibration associated with the Moon-forming impact^9^. In contrast, non-volatile lithophile elements are not affected by metal–silicate segregation and can track the entire accretion history of planets. The bulk of existing nucleosynthetic data for lithophile elements is based on nuclides that are not major planet-forming elements (that is, zirconium (Zr), strontium (Sr), titanium (Ti), neodymium (Nd), barium (Ba) and chromium (Cr)). Thus, a major step forward towards understanding the nature of the precursor material to terrestrial planets is developing a nucleosynthetic tracer that is a major planetary building block.

## Silicon as a novel nucleosynthetic tracer

We present a high-precision nucleosynthetic isotope analysis of silicon (Si), the most abundant refractory Solar System element. Type II supernovae are the principal nucleosynthetic source of all Si isotopes via stellar burning processes, with minor contributions from type Ia supernovae and asymptotic giant branch (AGB) stars (Methods)^12^. Thus, the bulk of Si in the Galaxy is synthesized by a ubiquitous stellar process as opposed to rare astrophysical environments such as type Ia or electron-capture supernovae. Although nucleosynthetic effects in Si isotopes were identified in refractory inclusions over three decades ago^13^, potential variations are small and require previously unattained analytical precision.

**Fig. 1:**
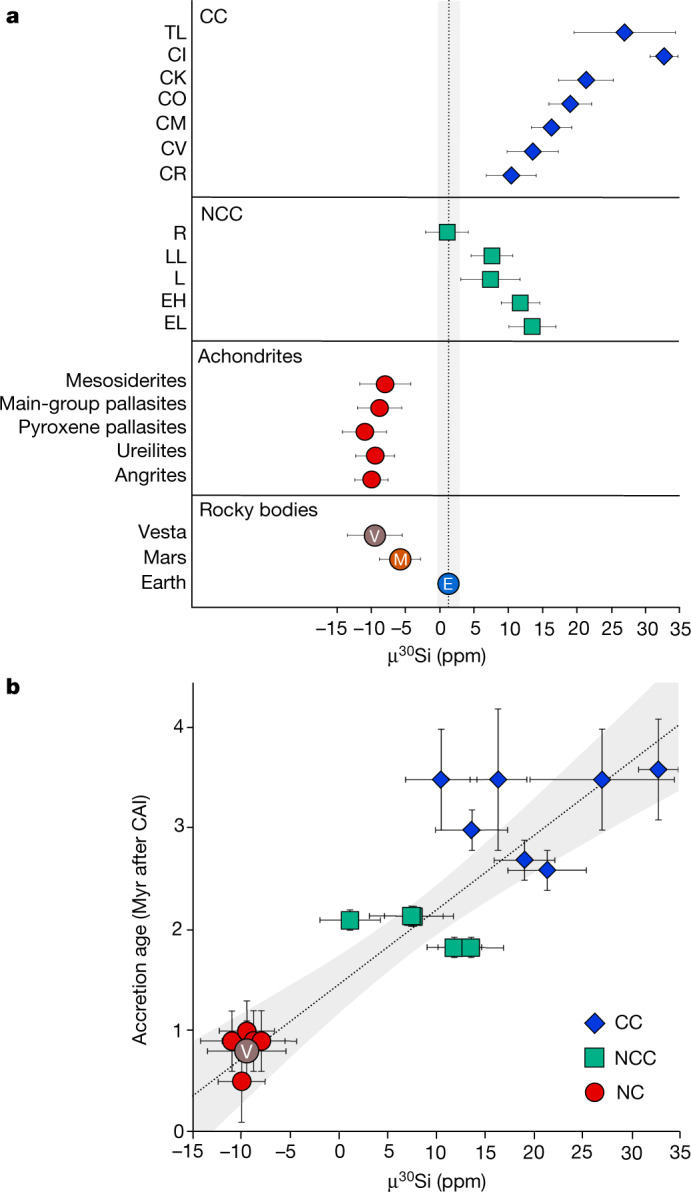
Mass-independent μ^30^Si data for bulk meteorites and a comparison with their accretion ages. **a**, Bulk meteorite μ^30^Si compositions. Error bars represent 2 s.e.m. of multiple analyses or 2 s.e. of the sample analyses. The vertical dashed line represents the mean terrestrial value derived from ten individual measurements of terrestrial basalt standards. The symbols M, E and V represent the composition of Mars, Earth and Vesta, respectively. **b**, μ^30^Si compositions versus their accretion ages. Meteorites are grouped into non-chondritic (NC), non-carbonaceous chondrites (NCC) and carbonaceous chondrites (CC). Error bars represent 2 s.e.m. The dashed line is a linear least-squares regression through all data points. Accretion ages in million years after calcium-aluminium-rich inclusion (CAI) formation and are taken from ref. ^27^. Source data

Improving the analytical precision by an order of magnitude over earlier studies, we analysed meteorites from Mars and Vesta as well as angrites, ureilites, pallasites and a mesosiderite, representing all major classes of inner Solar System achondrites. The primitive meteorites studied here include all main groups of carbonaceous and non-carbonaceous chondrites (Table 1 and Fig. 1a). The mass-independent silicon isotope data are reported as mass bias-corrected deviations from the NBS-28 standard in parts per million, using the μ-notation as follows: μ^30^Si = [(^30^Si/^28^Si)_sample_/(^30^Si/^28^Si)_NBS-28_ − 1] × 10^6^. Samples of differentiated planetesimals record identical μ^30^Si deficits relative to Earth’s mantle (μ^30^Si = −8.9 ± 1.4 ppm, 2 s.e., *n* = 29). By contrast, all non-carbonaceous chondrites apart from R chondrites are characterized by μ^30^Si excesses ranging from +7.4 ± 4.3 ppm to +13.5 ± 3.4 ppm. Carbonaceous chondrites are the most μ^30^Si-enriched materials, with μ^30^Si excesses from +10.4 ± 3.6 ppm to +32.8 ± 2.0 ppm. Mars has a Si isotope composition distinct from Earth with a μ^30^Si signature akin to differentiated planetesimals (μ^30^Si = −5.8 ± 3.0 ppm).

**Table 1:** Mass-independent μ^30^Si data for bulk meteorites

	μ^30^Si	2 s.e.	*n*
**Terrestrial planets**			
Earth	1.2	1.5	10
Mars	−5.8	3.0	5
**Differentiated meteorites**			
Angrites	−10.0	2.4	6
Vesta	−9.5	4.0	5
Ureilites	−9.5	2.8	2
Main-group pallasites	−8.8	3.2	8
Pyroxene pallasites	−11.0	3.2	2
Mesosiderites	−8.0	3.7	1
**Non-carbonaceous chondrites**			
L	7.4	4.3	1
LL	7.6	3.0	3
EH	11.8	2.8	3
EL	13.5	3.4	3
R	1.1	3.1	1
**Carbonaceous chondrites**			
CR	10.4	3.6	2
CV	13.6	3.7	3
CM	16.3	3.0	3
CO	19.1	3.1	2
CK	21.4	4.0	2
TL	27.0	7.4	1
CI	32.8	2.0	10

A summary of the μ^30^Si compositions for asteroidal and planetary bodies. The number of individual analyses comprising distinct samples or repeat analyses of the same sample is denoted by *n*.

**Extended Data Fig. 1:**
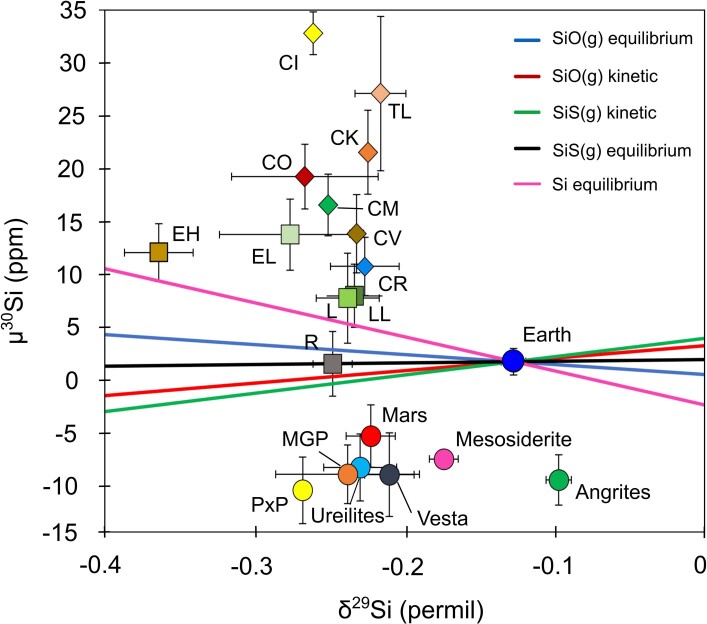
Triple Si isotopic systematics for a range of Solar System materials. Error bars represent 2 s.e.m. of multiple analyses or the 2 s.e. of the sample analyses if the group consists of one sample. Mass-dependent fractionation lines are calculated for all viable Si-bearing species assuming both kinetic and equilibrium fractionation laws. Kinetic [β = ln(m_1_/m_2_)/ln(m_1_/m_3_)] and equilibrium [β = [(1/m_1_)-(1/m_2_)]/[(1/m_1_)-(1/m_3_)] fractionation factors were calculated for Si, SiO, and SiS, where m_1_ = ^28^Si, ^28^SiO or ^28^SiS, m_2_ = ^29^Si, ^29^SiO, or ^29^SiS, and m_3_ = ^30^Si, ^30^SiO, or ^30^SiS. The expected δ^30^Si for a set of arbitrary δ^29^Si values is then calculated using the above fractionation factors for Si and Si-bearing molecules. The mass fractionation lines shown represent the difference in parts per million of the expected δ^30^Si values for each fractionation law from kinetic law used in this study.

As the μ^30^Si variability is relatively small, it may be an artefact of inappropriate correction for natural mass-dependent fractionation. Isotopic fractionation of Si under nebula conditions occurs as oxidized silicon oxide (SiO) or reduced silicon sulfide (SiS)^14^. Alternatively, Si may be fractionated in elemental form by its partitioning into the core during planetary differentiation^15^. We show in Extended Data Fig. 1 samples measured here plotted in three isotope μ^30^Si–δ^29^Si space (δ^29^Si = [(^29^Si/^28^Si)_sample_/(^29^Si/^28^Si)_NBS-28_ − 1] × 10^3^) alongside mass-fractionation lines calculated using fractionation laws for gaseous SiO_(g)_ and SiS_(g)_ as well as atomic Si. No natural fractionation law can account for the μ^30^Si variability across Solar System materials. In principle, the difference between the μ^30^Si composition of Earth and EH chondrites may reflect partitioning of Si into Earth’s core. However, the magnitude of fractionation needed to explain the μ^30^Si disparity (Δ^30^Si_BSE-EH_ = 0.46, where BSE is bulk silicate Earth) requires a Si concentration in Earth’s core of >50 wt% (ref. ^16^) exceeding current estimates (about 6% (ref. ^15^)). Cosmogenic effects could also impart μ^30^Si variability. However, based on the indistinguishable μ^30^Si compositions of ureilites and pallasites, which have contrasting average exposure ages of 17 ± 15 million years ago (Ma) and 104 ± 36 Ma, respectively^17,18^, we conclude that cosmogenic effects are negligible. Presolar SiC grains are an important Si reservoir and, given their highly anomalous compositions (μ^30^Si ≈ −2,700 (ref. ^19^ and Methods)), heterogeneous distribution of silicon carbide (SiC) grains could impart μ^30^Si variability. However, mass-balance calculations require an increase in the SiC concentration in the source of achondrites about 75-times higher than CI chondrites, which we consider exceedingly unlikely given that such variability is not observed across chondrites^20^.

Enstatite chondrites, and all chondrites apart from R chondrites, show μ^30^Si excesses relative to Mars and Earth. This is in stark disagreement with the notion that Mars and Earth were formed by collisional mergers between a mixture of asteroidal bodies from which chondritic meteorites are derived. Instead, Mars and Earth require a contribution from material with a μ^30^Si-depleted signature recorded only by differentiated planetesimals. Although the Si isotopic composition of R chondrites matches that of Earth, different oxygen isotope signatures^21^, as well as magnesium (Mg)/Si and aluminium (Al)/Si ratios^22^, do not permit R chondrite-like material as the sole precursor material to Earth. Thus, a significant implication of this work is that material akin to that of differentiated planetesimals constitutes major building blocks of terrestrial planets.

## The μ^30^Si achondrite–chondrite dichotomy

Several studies have shown that meteorites exhibit a fundamental isotopic dichotomy between non-carbonaceous and carbonaceous groups^2,3^. For example, non-carbonaceous achondrite and chondrite parent bodies record deficits in the nucleosynthetic composition of neutron-rich nuclides such as ^54^Cr, ^48^Ca and ^50^Ti whereas carbonaceous parent bodies record excesses, relative to Earth. This compositional dichotomy is interpreted as reflecting the spatial isolation of the inner and outer Solar System reservoirs by the rapid formation of Jupiter^23^. This dichotomy is not apparent in Si isotopes, which shows overlap in the μ^30^Si values of non-carbonaceous and carbonaceous chondrites. The major division observed in Si isotopes is between the parent bodies of non-carbonaceous differentiated meteorites and that of chondrite meteorites, indicating the existence of an achondrite–chondrite dichotomy as opposed to a non-carbonaceous and carbonaceous dichotomy.

The parent bodies of differentiated meteorites studied here accreted in the inner disk^24^ (<3 au) <1 Myr after Solar System formation^25,26^. Chondritic bodies accreted later, with model ages of 1.8 ± 0.1 Myr and 2.1 ± 0.1 Myr for enstatite and ordinary chondrites, and 2.6 ± 0.2 Myr to 3.6 ± 0.5 Myr for carbonaceous chondrites^27^. In contrast to non-carbonaceous meteorites, water-rich carbonaceous chondrites^28^ accreted in the outer disk, possibly associated with dust enrichment in a pressure trap beyond Jupiter’s orbit^24^ or with a primordial ice line situated several astronomical units from the Sun^29^. Figure 1b shows a clear relationship between the accretion age of these bodies and their μ^30^Si composition. The composition of the young (<1 Myr), inner protoplanetary disk recorded by differentiated planetesimals is characterized by a uniform depletion in μ^30^Si. We observe a shift in the μ^30^Si values between differentiated meteorites and non-carbonaceous chondrites that both accreted in the inner disk but at different times. As such, our results establish that the μ^30^Si composition of the inner disk evolved within about 2 Myr of Solar System formation. This compositional change is consistent with progressive admixing of a high-μ^30^Si CI-like outer Solar System dust component to the inner disk^4^. Thus, we interpret the μ^30^Si achondrite–chondrite dichotomy as a temporal feature, alleviating the need for spatial isolation of inner and outer disk reservoirs^30^.

**Fig. 2:**
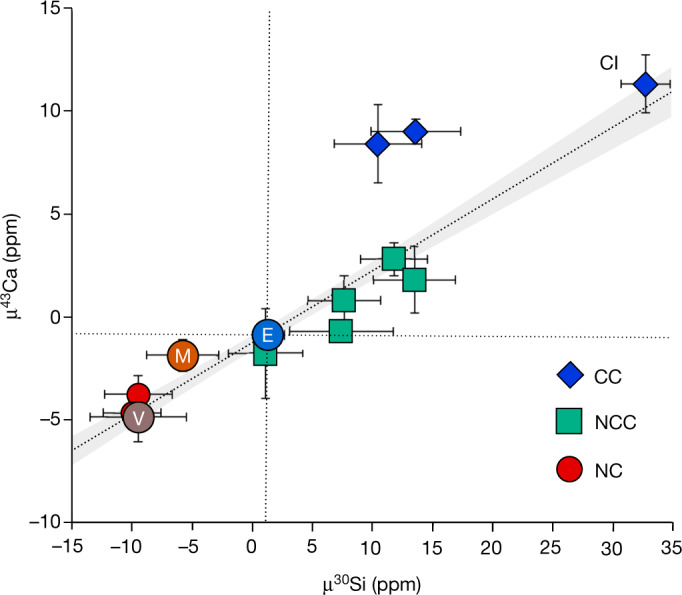
Multi-element isotope plot of μ^30^Si and μ^43^Ca values for bulk Solar System objects. Meteorites are grouped into non-chondritic (NC), non-carbonaceous chondrites (NCC) and carbonaceous chondrites (CC). Uncertainties for both μ^30^Si and μ^43^Ca are 2 s.e.m. The dashed line is a linear least-squares regression through inner-disk bodies and CI chondrites. The μ^43^Ca data and sources are available in Supplementary Table 2. Source data

We compare in Fig. 2 the μ^30^Si values of inner-disk bodies with their μ^43^Ca compositions, a nuclide also synthesized by stellar burning processes^31^. In detail, ^43^Ca as well as ^42^Ca and ^44^Ca that are utilized for internal normalization are predominantly formed by oxygen and Si burning in massive stars (Methods). Although ^44^Ca is produced by the decay of the short-lived ^44^Ti nuclide, it may be considered a product of oxygen and Si burning as ^44^Ti is synthesized this way before rapidly decaying to ^44^Ca (half-life (*t*_1/2_) = 60 yr). The μ^30^Si–μ^43^Ca values are strongly correlated (Fig. 2), a feature that is consistent with their predicted similar nucleosynthesis. Moreover, this observation suggests that the Solar System’s Si and ^43^Ca variability broadly reflects the unmixing of two nucleosynthetic components. This is in agreement with the recent proposal that the Solar System’s nucleosynthetic variability can be accounted for by destruction of interstellar dust in the inner disk resulting in the enrichment of slow-neutron-capture-process (*s*-process)-dominated stardust^32^. The ubiquitous μ^30^Si depletions recorded by early-formed bodies, including Mars, represent the Si isotope composition of the initial, thermally processed reservoir enriched in stardust. Progressive admixing of outer Solar System material to the inner disk by inwards drift results in a replenishment of the interstellar dust component, such that later-accreted inner-disk bodies such as ordinary and enstatite chondrites will record higher μ^30^Si compositions.

## Planetary accretion and isotope diversity

Mars is believed to represent a stranded planetary embryo formed by accretional collisions between planetesimals within the 3–5-Myr disk lifetime^33^. Current models predict that Mars’ precursor material consisted of a mixture of ordinary, enstatite and carbonaceous chondrites^34^ with minor contribution from material akin to angrites^35^. However, the μ^30^Si value of Mars (−5.8 ± 3.0 ppm) cannot be produced by mixing any combination of chondritic components. Instead, the similar μ^30^Si values of Mars and early-formed differentiated planetesimals indicate its formation by collisional mergers between these types of bodies. This is an outcome of differentiated planetesimals being available building blocks in the inner disk for a significantly greater fraction of Mars’ accretion period than chondrite parent bodies. Thus, Mars must have completed its growth within about 2 Myr to avoid incorporation of enstatite or ordinary chondrite-like material.

The uniform depleted μ^30^Si signal recorded by inner Solar System differentiated planetesimals suggests that this composition represents that of the disk material available to fuel the growth of proto-Earth. The μ^30^Si value of Earth is intermediate between non-carbonaceous achondrite and chondrite meteorites. Thus, the proto-Earth could have formed by collisional accretion of a mixture of non-carbonaceous achondrite and chondrite parent bodies after disk dissipation, requiring a chondritic contribution ranging from 28% to 73% of Earth’s current mass (calculated by admixing chondrite-type endmember compositions to reproduce the terrestrial value assuming invariant Si concentrations). This high chondrite fraction is inconsistent with Earth’s volatile element budget, including its noble gas^36^ and nitrogen^37^ inventory. Although volatile loss can occur via impacting bodies, this mechanism is not efficient enough to remove the required amounts of volatiles^38^. Moreover, the terrestrial noble gas inventory does not support impact-driven erosion of Earth’s primordial atmosphere^36^.

**Extended Data Fig. 2:**
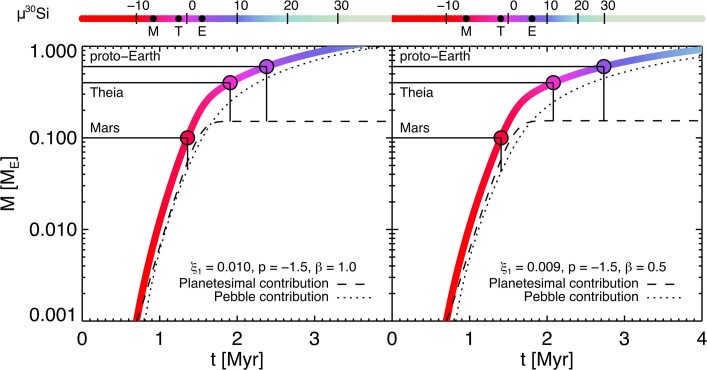
Two growth curves of Proto-Earth, Theia and Mars that comply with the μ^30^Si values for Mars and for the combined Theia-Proto-Earth system. The top bar shows the instantaneous value of μ^30^Si in the terrestrial planet zone. The planetary growth model is based on ref. ^11^ and includes both pebble accretion and planetesimal accretion in an evolving protoplanetary disk. We grow Earth to 0.6 M_E_ and an additional planet, Theia, to 0.4 M_E_ to complete Earth’s growth in a giant impact between the two. The planetesimals are placed in a narrow belt around 1.6 AU. The left panel shows a model where μ^30^Si increases linearly with time (as in Fig. 1b) and the pebble flux is 1.0% times the gas accretion rate onto the star after 1 Myr and then falls as t^ρ^ where ρ = −1.5. Here Mars forms within 1.5 Myr and proto-Earth within 2.5 Myr. Assuming instead a smaller pebble flux (0.9% of the gas flux) and a more abrupt admixing of CI-like material into the inner regions of the protoplanetary disk [t(μ^30^Si) = K*(μ^30^Si - μ^30^Si_0_)^β^ with β = 0.5 and K adapted to yield μ^30^Si = +10 at t = 2 Myr when the ordinary chondrite parent bodies form], the right panel shows that Earth formation within 3 Myr matches the Si isotopic composition of proto-Earth. This more abrupt transition in the composition of the inner protoplanetary disk yields a final accretion of material with a CI-like composition, in agreement with iron isotope measurements for Earth^8^. For each planet, the contribution from pebble and planetesimal accretion are indicated. For example, for Mars (0.1 M_E_) pebble and planetesimal accretion contribute equally (0.05 M_E_) to the final mass of the planet.

The observed secular change in the μ^30^Si value of inner-disk material is in line with previous suggestions that terrestrial planets accreted a mixture of inner-disk material akin to ureilites and pristine inwards-drifting CI chondrite-like outer Solar System pebbles^4,8,11^. We explore in Methods whether the μ^30^Si composition of Mars and proto-Earth can be reproduced if their growth occurred by a combination of collisions and pebble accretion during the disk lifetime. Irrespective of the model parameters, our results require that Mars and the proto-Earth completed their growth within the 5-Myr disk lifetime (Extended Data Fig. 2). The fraction of CI-like pebbles accreted by proto-Earth and Mars corresponds to 26 ± 9% and 10 ± 12%, respectively (calculated by admixing CI-like pebbles to an achondrite composition to reproduce the μ^30^Si values of Earth and Mars assuming invariant Si concentrations). In contrast to collisional accretion, where the volatile inventory of impacting material is delivered to the growing body, pebbles are thermally processed and devolatilized while falling towards the protoplanet’s surface. Conditions for the thermal processing of infalling material are self-sustained by the rapid accretion of pebbles, leading to high-accretion luminosities that support a growing gas envelope around the planetary core^39–41^. This mechanism can decouple the refractory and volatile element budget of the accreting material and, thus, the high fraction of CI-like material in Earth’s precursor inferred here does not violate the volatile element inventory of Earth.

**Fig. 3:**
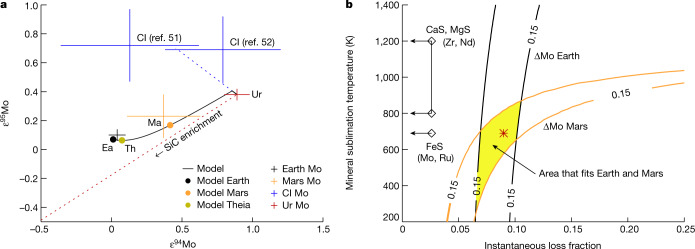
Mo isotope evolution of a planet experiencing thermal envelope processing during pebble accretion. **a**, The dotted blue line indicates the addition of CI material to a starting material akin to ureilites, and the dotted red line indicates the enrichment of SiC by loss of non-SiC-hosted Mo (that is, sulfide), which have a composition complementary to SiC that when combined yields the CI ε^94,95^Mo value. The crosses indicate two measurements of CI chondrites^51,52^, Earth (Ea), Mars (Ma) and ureilite (Ur) (ref. ^3^). The black line shows the model evolution of (ε^94^Mo, ε^95^Mo), with the masses of Mars, Theia (Th) and Earth indicated as full circles. The survival of SiC grains leads to an enhancement of SiC in the planet that agrees with the *s-*process excess of Mars and Earth in Mo. **b**, Contours of 0.15 ε-unit radial distance ΔMo between model and data points for (ε^94^Mo, ε^95^Mo) of Earth (black) and Mars (orange) as a function of the fraction of Mo lost (*x* axis) and the sublimation temperature (*T*_sub_; *y* axis). The loss fraction (instantaneous loss fraction when *T* > *T*_sub_) and sublimation temperature agree well with both Earth and Mars data in the yellow area. The acceptable range of loss fractions corresponds to a 7–11% enrichment of SiC. The red point marks the conditions that reproduce the model plotted in **a**. We indicate the sublimation temperature of FeS to highlight a possible mineral carrier of Mo as well as that of CaS and MgS, which are important hosts of Zr and Nd. It is noted that the loss fraction of Mars rises rapidly as the mineral sublimation temperature approaches 1,000 K, the approximate maximum temperature of Mars during accretion.

**Extended Data Fig. 3:**
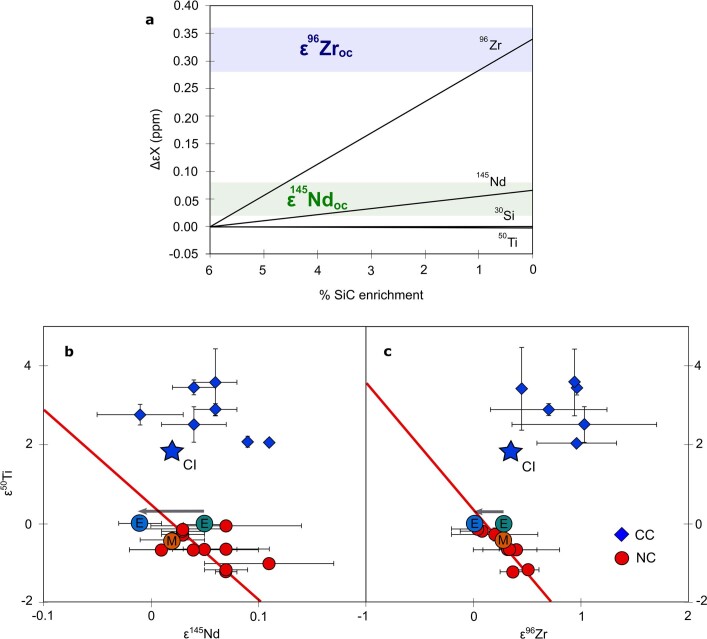
The effect of enriching Earth in SiC by sublimation of a component that carries the complementary isotopic composition of SiC grains. **A**) Black lines indicate the effect of SiC enrichment on the μ^30^Si, ε^50^Ti, ε^96^Zr, and ε^145^Nd composition of Earth. Note that Si is not affected by SiC enrichment. Green and blue bands represent 2 standard error intervals of the Nd and Zr composition of ordinary chondrites. The ordinary chondrite ε^50^Ti composition is off the scale (ε^50^Ti_OC_ = −0.66) (ref. ^3^). The Si and Ti isotope composition of Earth is insensitive to SiC enrichment by thermal processing. Potential Nd and Zr carrier mineral phases (CaS, MgS) that may be destroyed, and their sublimation temperatures are indicated in Fig. 3. Note that % SiC enrichment (x) relates to loss fraction (y) by the following equation y = 0.9432× + 0.0463. **B**) The effect of 5–7% enrichment of SiC grains by thermal processing on the ε^50^Ti-ε^145^Nd composition of Earth. **C**) The effect on the ε^50^Ti-ε^96^Zr composition of Earth. Red lines are linear least squares regressions through non-carbonaceous bodies. Earth modelled without envelope processing is in green and Earth after envelope processing (as measured) is in blue. The arrows indicate the effect of envelope processing on the isotopic composition of Earth.

Thermal processing of pebbles in the hot planetary gaseous envelope (>1,000 K (ref. ^40^)) surrounding an accreting protoplanet can further impart nucleosynthetic variability by selective destruction of volatile carriers, in a similar fashion as proposed for thermal processing of dust in the inner protoplanetary disk^2,32^. Envelope processing is modelled to occur when a protoplanet reaches about 0.02 *M*_E_ (ref. ^11^) and, as such, this mechanism is only relevant for Earth and Mars. The nucleosynthetic composition of achondrite and chondrite parent bodies, which owe their growth to the streaming instability, is not affected by envelope processing. Thus, these bodies cannot be used to trace the precursor material to Earth and Mars, especially for nucleosynthetic tracers sensitive to envelope processing. Earth is enriched in *s*-process matter relative to most inner Solar System bodies, a feature used to argue that an important planetary building block is unsampled, *s*-process-enriched inner Solar System material^3,42^. This conclusion is not supported by the Si isotope data presented here. Thus, we explore the role of envelope processing in modifying the Mo isotope composition of Earth and Mars as this element is widely used to infer their accretion history. We adopt the model of ref. ^32^, where the Solar System’s *s*-process variability represents variable unmixing of two dust reservoirs, namely, homogenized interstellar dust and an *s*-process-dominated stardust component that, when combined, reflect the solar composition. Sulfides (FeS, MgS and CaS) are an important reservoir for Mo (ref. ^43^) as well as other trace elements such as Zr and Nd in reduced chondrites^44,45^. Importantly, astronomical observations suggest that >90% of the sulfur budget in young disks is hosted in refractory sulfides, indicating that these minerals are ubiquitous in protoplanetary disks^46^. Sulfide destruction by envelope processing at expected temperatures ≳700 K will progressively enrich the accreted material in the *s*-process-dominated stardust component. Figure 3 simulates the Mo isotope evolution of a planet experiencing sublimation of a component carrying the complementary isotopic composition of the *s*-process-dominated stardust during pebble accretion. We find that the *s*-process excess of Mars and Earth can be reproduced if about 10% of Mo residing in the homogenized interstellar dust component is lost during planetary envelope processing, alleviating the need for a missing inner Solar System reservoir. The loss fraction refers to the instantaneous fraction of Mo lost when the envelope temperature is above the sublimation temperature of sulfides. An additional contribution of planetesimals (not affected by envelope processing) to Earth will increase the necessary loss fraction from pebbles by a factor 1/*f*_peb_, where *f*_peb_ is the pebble contribution fraction (25% planetesimal contribution thus gives 13% loss fraction of Mo). Using the constraints from Fig. 3, we show in Extended Data Fig. 3 that this enrichment of *s*-process-dominated stardust during envelope processing can reproduce the terrestrial *s*-process excess in Zr and Nd relative to inner-disk bodies. After correction for envelope processing effects, Earth’s composition lies between that of non-carbonaceous achondrites and CI chondrites (Extended Data Fig. 3), consistent with the Si isotope data. Thus, Earth’s *s*-process excess relative to achondrite and chondrite parent bodies is a hallmark of its different accretion history, namely, pebble accretion as opposed to streaming instability.

**Extended Data Fig. 4:**
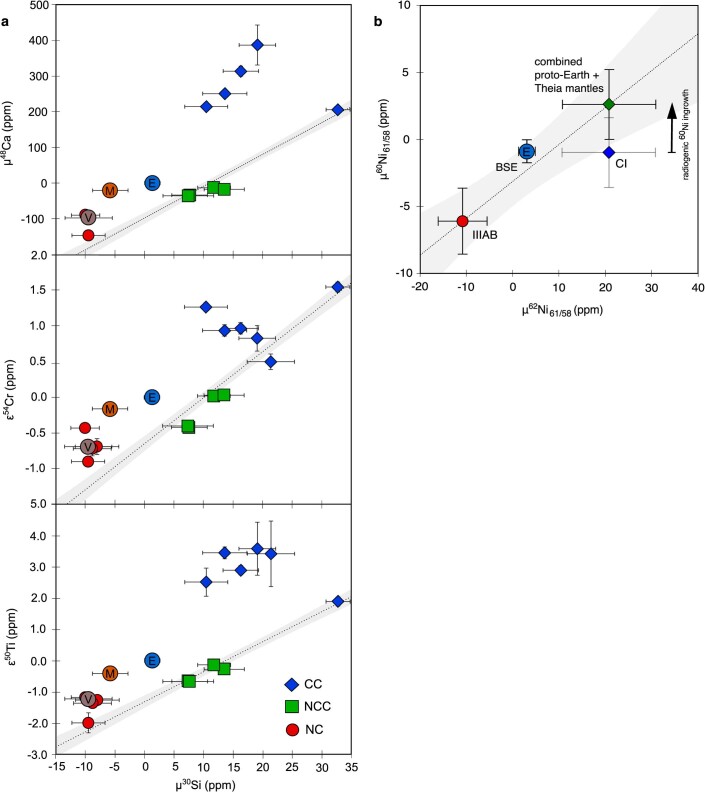
Multi-isotope plots showing the µ^30^Si values plotted against µ^48^Ca, ε^54^Cr and ε^50^Ti (A) as well as a µ^60^Ni vs µ^62^Ni diagram (B). CC, carbonaceous chondrites, NCC, non-carbonaceous chondrites, NC, non-carbonaceous achondrites, E, Earth, V, Vesta. Errors are 2 s.e.m. The dashed lines are linear least squares regressions through ureilites, NCC, and CI chondrites. For µ^48^Ca and ε^50^Ti deviations from the correlation lines may reflect variable addition of refractory inclusions and/or lack of incorporation of a volatile carrier depleted in these isotopes. For ε^54^Cr additional scatter may exist due to incorporation of ^54^Cr-rich supernovae oxide grains. The Ni isotope diagram shows that the terrestrial mantle lies on a mixing line between IIIAB and the combined mantles of proto-Earth and Theia (prior to equilibration with Theia’s core) when radiogenic ingrowth of ^60^Ni is accounted for (see Methods). µ^48^Ca data is from refs. ^4^ and ^35^. ε^54^Cr and ε^50^Ti data is from a compilation by ref. ^3^. and references therein. Data sources for the Ni isotope diagram are detailed in the Methods section (Iron-nickel isotope evolution simulations).

Extended Data Fig. 4 shows the μ^30^Si values of various meteorite parent bodies plotted against their ^54^Cr, ^48^Ca and ^50^Ti compositions. In contrast to the μ^30^Si–μ^43^Ca Solar System correlation line, more scatter exists, possibly reflecting the presence of multiple carriers of anomalous neutron-rich isotopes. For example, ^48^Ca and ^50^Ti are sensitive to nugget effects from refractory inclusions, whereas ^54^Cr-rich supernovae-derived spinels^47^ may induce additional ^54^Cr variability. Moreover, ^54^Cr-poor and ^50^Ti-poor compositions have been identified in the aqueous alteration phases of primitive carbonaceous chondrites^2,48^, implying the existence of yet another carrier of anomalous Cr and Ti. In contrast to Earth and Mars, which owe a significant part of their growth to pebble accretion, chondrite and achondrite parent bodies are understood to have formed by streaming instability at the ice line^49^. Thus, the different accretion mechanism of Earth and Mars, and in particular the presence of hot planetary gaseous envelopes during their accretion, may result in heterogeneous incorporation of anomalous neutron-rich isotope carriers and, hence, decoupling between the μ^30^Si and ^54^Cr–^48^Ca–^50^Ti values.

**Fig. 4:**
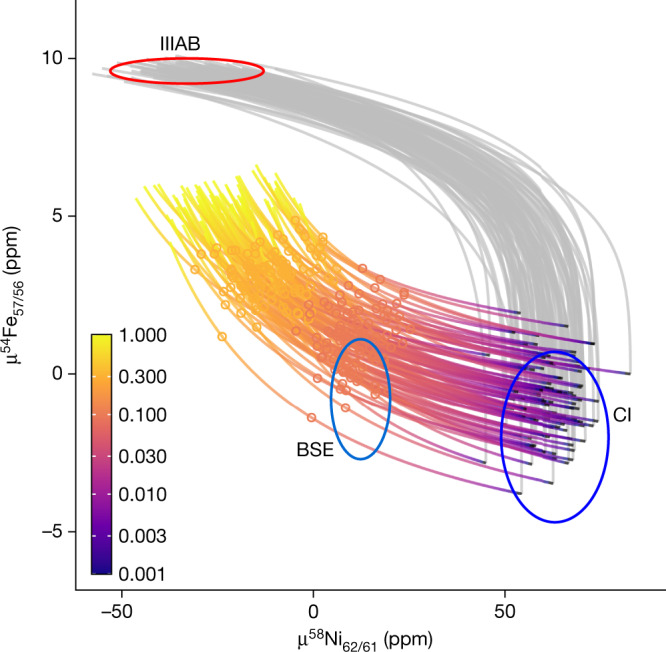
Monte Carlo simulations of Fe–Ni isotope evolution of the terrestrial mantle during concurrent accretion and core formation in μ^54^Fe_57/56_–μ^58^Ni_62/61_ space. Ellipses represent 2 s.e. intervals for the measured BSE, IIIAB and CI isotope compositions. Ni and Fe isotope data are from refs. ^8,53–55^. The grey lines represent the evolution of the proto-Earth mantle during pebble accretion. The coloured lines represent various outcomes of the Moon-forming giant impact depending on the fraction of the Theia’s core that equilibrated with Earth’s mantle during impact. The equilibration degree is shown by the colour scale and circles highlight 10% and 30% of equilibration with Theia’s core.

The nucleosynthetic isotope composition of Earth’s mantle is CI-like for the siderophile element iron, which is interpreted as reflecting rapid differentiation of the proto-Earth during the disk lifetime^8^. However, the composition of Earth’s mantle is not CI-like for the siderophile element nickel (Ni), which conflicts with this model. The final mass of Earth includes a contribution from Theia, which collided with the proto-Earth to form the Earth–Moon system. Thus, partial equilibration of Theia’s core with the proto-Earth can modify the final composition of the terrestrial mantle. Ni is very siderophile under the low-pressure conditions associated with accretion of proto-Earth by pebble accretion but becomes increasingly lithophile at the high-pressure conditions expected during the Moon-forming impact^50^. Figure 4 shows Monte Carlo simulations that model the iron (Fe) and Ni isotope evolution of Earth’s mantle during pebble accretion and, subsequently, following the giant impact for different degrees of equilibration with Theia’s core. Accepting a mass of 0.6 *M*_E_ and 0.4 *M*_E_ for the proto-Earth and Theia, respectively^11^, the Fe and Ni isotopic composition of Earth’s mantle can be reproduced under a relatively low level of equilibration with Theia’s core (<20%). We note that the Ni isotope composition of Earth’s mantle is not on a mixing relationship between achondrites (that is, group IIIAB iron meteorites) and CI chondrites on a μ^60^Ni–μ^62^Ni diagram. Earth’s and Theia’s mantles at the end of pebble accretion at about 2.5 Myr are inferred to have CI-like Ni isotopes and a Fe/Ni ratio of about 193 from the model described in Fig. 4 (Methods). This superchondritic Fe/Ni ratio will result in modest radiogenic ingrowth of ^60^Ni from the short-lived ^60^Fe nuclide (*t*_1/2_ ≈ 2.6 Myr) such that the Ni isotope composition of the combined mantles of Earth and Theia before partial equilibration with Theia’s core aligns on a mixing relationship with Earth’s modern mantle and group IIIAB iron meteorites (Extended Data Fig. 4d). Thus, the Ni isotope composition of the terrestrial mantle and, in particular the presence of radiogenic ^60^Ni, is consistent with rapid formation of the proto-Earth by pebble accretion within the disk lifetime.

## Conclusions

The cosmochemistry of Si, a major planetary component, provides a novel perspective on the accretion history of terrestrial planets, emphasizing the role of early-formed, inner Solar System differentiated asteroids as major planetary building blocks. Importantly, the Si nucleosynthetic composition of asteroidal and planetary bodies is impervious to admixing of refractory presolar SiC grains in contrast to minor planetary components such as Mo and Zr. Planet formation by pebble accretion predicts significant thermal processing of the accreted material, as opposed to planetesimal accretion and planet formation by the streaming instability and collisional growth, respectively. Thus, the primary composition of tracers such as Mo and Zr can be modified by secondary, volatility-driven processes during planetary growth by pebble accretion. Similarly, the nucleosynthetic composition of Earth’s mantle for siderophile elements can be modified by equilibration with Theia’s core during the Moon-forming impact. A final implication of this work is that comparison of various nucleosynthetic tracers without careful consideration of their geochemical behaviour, the nature of their carrier phases (that is, volatile versus refractory) and, lastly, their nucleosynthetic origin may not provide meaningful information on the nature of material precursor to terrestrial planets.

## Methods

### Sample preparation and isotope analysis

Sample digestion was performed using an alkali fusion technique^56^. Approximately 5–7 mg of powdered sample from an initial mass of about 1 g for chondrites and 15–30 mg for achondrites was mixed with sodium hydroxide (NaOH) pellets (>99.98% purity) in silver crucibles (99.99% purity) with a NaOH/sample ratio of about 30. The silver crucibles were then heated in a furnace at 720 °C for 13 min, producing a hyperalkaline liquid in which the silicate portion of the sample breaks down. The alkaline sample solution was then cooled at room temperature to form a metastable silicate complex. After cooling, 5 ml of 18.2 MΩ cm^−1^ water was added to the crucibles before 15-min ultrasonic treatment. The sample solution was then transferred to a Savillex Teflon beaker. This step was repeated three times until a final solution of 15 ml was made to ensure complete transfer of the sample. The solutions were acidified using 7 M nitric acid (HNO_3_) to digest any metals that are unaffected by NaOH fusion. We note that some laboratories favour the use of hydrochloric acid (HCl) to avoid additional ^14^N^16^O^+^ polyatomic interference on ^30^Si. We found that sample-standard mismatches in HCl molarity induce a greater matrix effect than HNO_3_ when performing isotope analyses on our instrument (δ^30^Si offset of 0.1‰ requires an approximately 0.02-M mismatch for HCl and an approximately 0.32-M mismatch for HNO_3_). Therefore, we use HNO_3_ as acid in our procedures given that the final solutions are not evaporated to dryness before isotopic analysis, which prevents us from matching the molarity of the sample and standard by conventional means. After acidification, the samples were ultrasonicated for 20 min and shaken vigorously halfway. A further 25 ml of 18.2 MΩ cm^−1^ water was added to the samples to produce a final volume of about 40 ml. Such large volume dissolutions aid in preventing precipitation of Fe hydroxides, which have been shown to preferentially adsorb light Si isotopes^57^. Finally, the pH of the solutions was adjusted to 2–3 before cation exchange chromatography as monosilicic acid (H_4_SiO_4_) is most stable from polymerization at this pH range^58^.

Following dissolution of the samples, purification of Si was achieved by cation exchange chromatography^56^. Columns were filled with 3 ml BioRad DOWEX 50W-X12 (200–400 mesh) cation exchange resin in H^+^ form. The resin was precleaned by rinsing several times, alternating between 6 M HCl, 7 M HNO_3_ and 18.2 MΩ cm^−1^ water. The resin was then preconditioned to a neutral pH using about 20 ml 18.2 MΩ cm^−1^ water before sample loading. The total amount of Si loaded onto the columns was about 150 μg for samples and about 300 μg for quartz-sand standard NBS-28. Silicon was eluted with 6 ml of 18.2 MΩ cm^−1^ water (pH neutral), while all cationic species are retained by the resin. In preparation for mass spectrometry, the eluted sample and standard solutions were adjusted to a final molarity of 0.5 M HNO_3_. Following the approach of ref. ^59^, the solutions were also doped with a sulfate solution to a fixed S/Si ratio of about 5. Sulfur doping was found to be critical in minimizing matrix effects between sample and standard solutions imparted by SO_4_^2−^ ions that are not retained by the cation exchange resin.

Silicon isotope measurements were performed using a Thermo Scientific Neptune Plus multi-collector inductively coupled plasma mass spectrometer (ICP-MS) at the Centre for Star and Planet Formation, University of Copenhagen. Samples of about 4-ppm Si were aspirated into the plasma source in 0.5 M HNO_3_ using an Apex-Q (Elemental Scientific) with an ACM Nafion fluoropolymer membrane desolvation module and and approximately 100 μl min^−1^ SeaSpray nebulizer. Instrumental sensitivity at this uptake rate was typically 30 V ppm^−1^ in high-resolution mode. The high tolerance of the SeaSpray nebulizer to total dissolved solids mitigates against blockages in the nebulizer by precipitation of silica. The dry plasma conditions generated by this set-up reduce the isobaric effects of interfering molecular species. Samples were measured against the quartz sand standard NBS-28 (NIST RM8546) using standard sample bracketing to correct for instrumental mass bias drift. The Faraday cup collecting the ^28^Si beam was equipped with a 10^10^-Ω amplifier, whereas ^29^Si and ^30^Si were collected in cups connected to 10^11^-Ω amplifiers. This configuration allowed for measurement of ^28^Si at signal intensities of at least 100 V. Signal intensities of the sample and standard were matched to within 5%. The instrument was operated in high-resolution mode with a minimum mass resolving power of about 11,000 (*m*/Δ*m* as defined by the peak edge width from 5–95% full peak height) to effectively resolve isobaric interferences on the measured isotopes of Si. A new high-resolution slit (27 μm) was installed before each measurement session, which typically showed signs of degradation after one week. Mass resolving power was monitored carefully on a 24-h basis to ensure the resolution of the source slit remained suitably high for analysis. A typical Si isotope session consisted of 2 or 3 samples, each measured either 5 times for 100 cycles with a 16.6-s integration time or 10 times for 100 cycles with an 8.39-s integration time, corresponding to approximately 15 ml of solution for a full sample analysis. The NBS-28 standard was also measured in same fashion as the samples before and after each sample measurement. Between each measurement, a blank acid solution identical to that used for sample dilution was measured for 25 cycles of 8.39 s. On-peak background intensity was typically <0.1% of the total signal and did not vary significantly between sessions. A peak centre was performed at the start of each session, with no peak centres during the session as peak drift was negligible.

**Extended Data Fig. 5:**
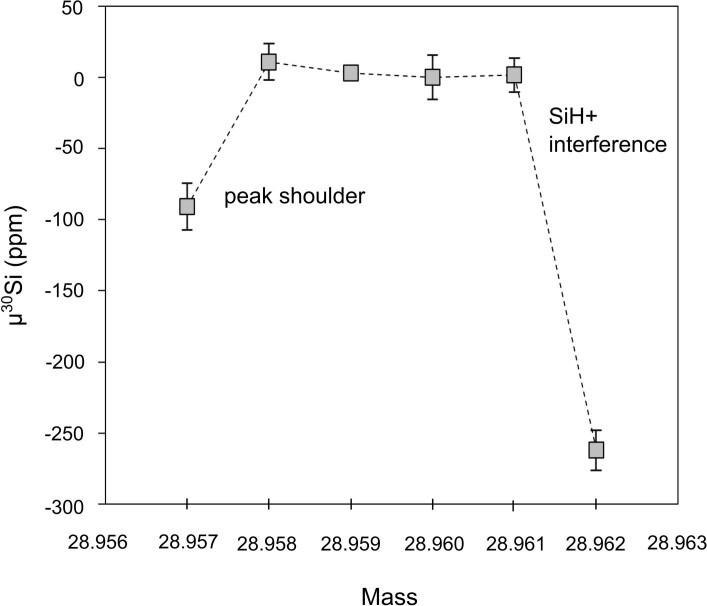
Plot showing the variation of the μ^30^Si value across the peak flat to illustrate the proximity of the peak edge and the SiH+ interference. Error bars represent 2 s.e. of the sample analyses. This example shows that there is a 4 millimass unit plateau where the measurements can be positioned, which is typically at mass 28.960.

As with other elements in this range of the mass spectrum, there are a host of potential molecular interferences on the isotopes of Si, such as ^12^C^16^O^+^ and ^14^N_2_^+^ on ^28^Si, ^28^Si^1^H^+^ on ^29^Si, and ^14^N^16^O^+^ on ^30^Si. As the interferences are slightly heavier than the respective isotope of interest, measurement on the left side of the Si peak shoulder allows the measurement of an interference-free analyte beam. The high-resolution mode (*m*/Δ*m* > 11,000) utilized in this study provided sufficient mass resolving power to eliminate isobaric interferences on the Si beams. For example, resolving the most problematic interferences ^28^Si^1^H^+^ (28.984762 amu) on ^29^Si (28.97649 amu) and ^14^N^16^O^+^ (29.99799 amu) on ^30^Si (29.97377 amu) requires a mass resolving power of *m*/Δ*m* = 3,500 and *m*/Δ*m* = 1,240, respectively. To determine the optimal mass position for isotopic analysis, shoulder tests were performed whereby we measured ^29^Si/^28^Si and ^30^Si/^28^Si ratios at a range of mass positions on the low-mass side of the Si peak to calculate δ^30^Si and μ^30^Si. This procedure was performed before each analytical session to ensure measurement of an interference-free beam. An example of μ^30^Si values independently calculated for a range of axial masses (Extended Data Fig. 5) shows a mass range of about four millimass units free of interfering species. The typical mass position selected for analysis was 28.960 as the effects from isobaric interferences were insignificant at this mass. In particular, measurement at this mass position ensures that data acquisition is not affected by the ^28^Si^1^H^+^ interference, appearing 0.008 amu after initiation of the Si peak. All data reduction was conducted offline using the Iolite data reduction software^60^. Background intensities were interpolated using a weighted linear spline, and changes in mass bias with time were interpolated using a smoothed cubic spline.

During separation of Si by cation exchange chromatography, several additional anionic species may pass through the column. Consequently, distinct chemical differences in the Si separates between meteorite groups may cause mass bias between the different objects analysed. Matrix effects imparted by SO_4_^2−^ ions that are not retained by the cation exchange resin are suppressed by doping samples and standards with a sulfur solution; however, other species such as PO_4_^−^ may be problematic. To this effect, we determined the concentration of residual impurities in the Si fraction using an iCAP ICP-MS located at the Centre for Star and Planet Formation, using multi-element calibrated reference solutions. We determined the concentration of impurities for four samples representing the different sample matrices investigated in our study, namely, the BCR-2 terrestrial standard, the Orgueil carbonaceous chondrite, the QUE 97014 eucrite and the Krasnojarsk pallasite. No impurities above percent level relative to Si were detected, with Na, P, Cr and Mo being the only impurities present above 0.1% (Extended Data Table 1). To assess the effect of these impurities on our measurements, NBS-28 standard solutions doped with 2% Na, P, Cr and Mo (relative to Si) were measured using the same analytical approach employed for all samples. This level of contaminants is 3–10 times greater than observed in our samples. No measurable effects were detected on the δ^29^Si or μ^30^Si values within the uncertainty of our measurements (Extended Data Table 2) and, thus, we conclude that the level of impurities present in our sample is negligible.

**Extended Data Fig. 6:**
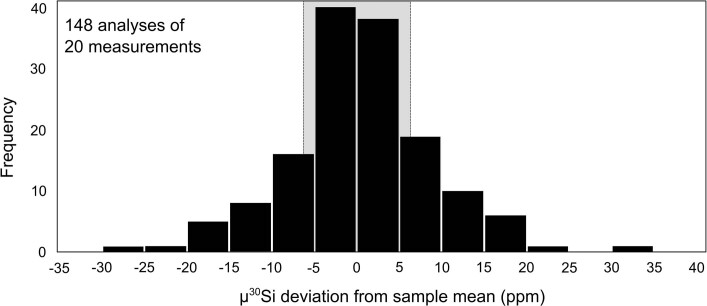
Histogram plot of μ^30^Si deviations from the sample mean for individual analyses of 10 terrestrial standard and 10 Orgueil samples. The grey bar represents the 2 standard error (2 SE) of 6.4 ppm.

To assess the long-term reproducibility of our measurements, the deviation of individual μ^30^Si measurements from the mean μ^30^Si value of the sample (as reported in Supplementary Table 1) is calculated for all terrestrial standard and Orgueil measurements, following an approach similar to that utilized by ref. ^61^. The included samples were measured over a 19-month period and are therefore expected to give an accurate prediction of long-term external reproducibility. Extended Data Fig. 6 shows a histogram plot of μ^30^Si deviations from the sample mean for 10 terrestrial standard and 10 Orgueil analyses, which corresponds to a total of 148 individual analyses. The histogram does not include statistical rejection of measurements, but only manual rejection owing to non-statistical reasons such as abrupt changes in sample uptake or samples running out of solution towards the end of an analysis. The μ^30^Si deviations show a normal distribution with a 2 s.d. external precision of 17.5 ppm, which corresponds to a 2 s.e. of 6.4 ppm given that each sample was analysed on average 7.4 times (20 samples for a total of 148 individual analyses). The precision of individual samples is reported as 2 s.e. of the sample mean in Supplementary Tables 1 and 2 and s.e. of the group mean in Table 1. The errors reported in Table 1 include distinct samples or repeat measurements of the same sample. The average 2 s.e. of all samples measured in this study is 5.4 ppm (*n* = 72). Hence, the internal and external reproducibility of sample measurements are comparable to within 1 ppm. In addition, the sample set used in the above calculation of external precision includes multiple digestions of the same sample as well as samples with significantly different matrices. As the 2 s.e. external precision of our dataset is comparable to the precision of individual sample analyses, we conclude that no additional source of uncertainty is introduced through sample dissolution or chemical separation of Si.

### Mass-dependent Si isotope composition of meteorites

**Extended Data Fig. 7:**
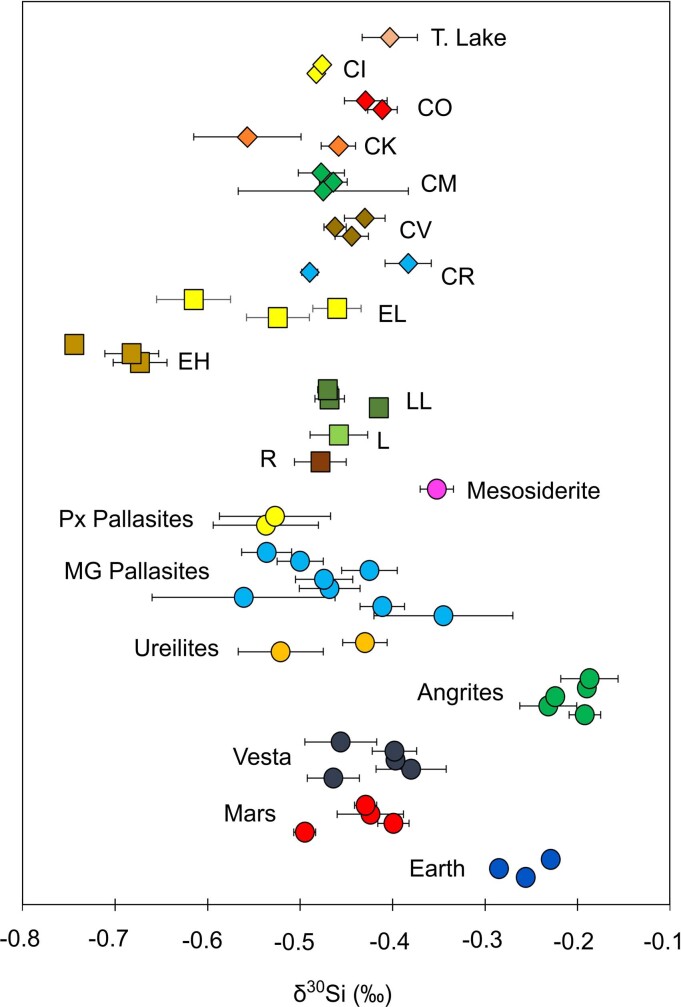
Mass-dependent δ^30^Si data for bulk meteorites. Error bars represent 2 s.e. of the sample analyses or the 2 s.e.m. of multiple analyses.

Bulk-rock mass-dependent Si isotope data are reported as δ^30^Si values for all measured meteorites in Supplementary Table 1 and Extended Data Fig. 7. The long-term reproducibility of our measurements was evaluated using mass-dependent isotopic compositions of three terrestrial basalt standards: BHVO-2, BIR-1 and BCR-2. Isotopic analysis of these terrestrial rock standards includes multiple dissolutions of the same rock powder, as well as chemical purification of Si performed in several independent sessions over a 19-month period. The standards were measured under the same analytical conditions as the meteorites and repeated over many sessions. Repeat measurements of BHVO-2 returned an average δ^30^Si of −0.26 ± 0.02 (2 s.e., *n* = 6), in good agreement with previously reported values. Similarly, basalts BIR-1 (δ^30^Si = −0.29 ± 0.03, 2 s.e., *n* = 1) and BCR-2 (δ^30^Si = −0.22 ± 0.02, 2 s.e., *n* = 3) also returned an average value within the range of values determined by other laboratories.

### Comparison with published Si isotope data

**Extended Data Fig. 8:**
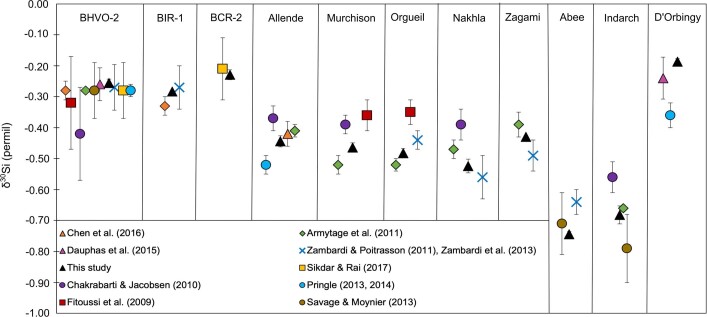
Comparison of mass-dependent δ^30^Si data for a range of well-studied standards and bulk meteorites. Error bars represent 2 s.e. of the sample analyses or the 2 s.e.m. of multiple analyses.

The only previous study of mass-independent Si isotope data was unable to resolve μ^30^Si variability from the terrestrial composition for all bulk meteorites apart from angrites^62^. The lack of variability in their study is attributed to insufficient analytical precision (about 30 ppm, 2 s.e.). The reported isotopic composition for angrites of μ^30^Si = −36 ± 20 ppm, however, is in agreement with the value of μ^30^Si = −10.0 ± 2.2 ppm reported in this study. Given that there are no precise mass-independent Si isotope data for meteorites, mass-dependent isotope values provide the best means for inter-laboratory comparison. Our δ^30^Si values for a range of well studied samples are in excellent agreement with those in the literature^16,63–71^, demonstrating the robustness of the analytical techniques used in this study (Extended Data Fig. 8).

### Mass-independent ^43^Ca isotope composition of meteorites

We compare the nucleosynthetic variability of ^30^Si and ^43^Ca to assess the consistency of our model. This comparison is justified by their production in similar nucleosynthetic environments and, thus, some co-variance is to be expected. The μ^43^Ca values for a range of Solar System materials are available in Supplementary Table 2. Some of these data are published values^72,73^ and are indicated in Supplementary Table 2. Additional μ^43^Ca data were obtained following the same analytical protocol as described in previous work^72,73^. The ^43^Ca isotope data are corrected for mass bias assuming kinetic fractionation and are reported as μ^43^Ca values, denoting mass-independent isotopic deviations of a sample relative to the SRM 915b standard: μ^43^Ca = [(^43^Ca/^44^Ca)_sample_/(^43^Ca/^44^Ca)_SRM915b_ − 1] × 10^6^.

### Nucleosynthesis of Si and Ca isotopes

The main Si isotope ^28^Si is significantly more abundant than ^29^Si and ^30^Si by virtue of ^28^Si being the most abundant product of the oxygen burning process in massive stars. The other two stable isotopes, ^29^Si and ^30^Si, exist primarily owing to hydrostatic oxygen and neon burning processes in massive stars and by explosive burning in the terminal stages of their evolution as type II supernovae^12,31^. In the case of low- to intermediate-mass stars, Si isotopes are produced in the AGB phase. In this thermally pulsing AGB phase, Si-bearing molecules condense in the outflows of the star to form grains (for example, SiC) that will preserve the Si isotopic ratios at the time of their formation. On the basis of the observation that ^29^Si/^30^Si isotope ratios in presolar SiC grains closely mimic the galactic chemical evolution line for Si isotopes, the AGB phase does not appear to significantly modify the galactically inherited Si isotope ratios^74^. Similarly, the production of Si isotopes in type Ia supernovae is negligible relative to their principal production during hydrostatic and explosive burning processes in massive stars^12^.

In the analysis of our data, we compare the μ^30^Si and μ^43^Ca compositions of meteorites owing to the similar nucleosynthetic heritage of ^30^Si, ^43^Ca and the isotopes involved in normalization for their respective μ values (^28^Si and ^29^Si for μ^30^Si, ^42^Ca and ^44^Ca for μ^43^Ca). As for all three isotopes of Si, ^42^Ca and ^43^Ca are primarily produced by both hydrostatic and explosive oxygen burning in massive stars with some contribution from neutron capture in the stars’ mantle^31^. The ^44^Ca isotope is a decay product of the extremely short-lived radionuclide ^44^Ti (*t*_1/2_ = 60 yr) that is also produced during oxygen and Si burning processes. Hence, the co-production of Si and the mentioned Ca isotopes by comparable nucleosynthetic processes can account for their strong co-variance over, for example, the more anomalous ^48^Ca, which is produced in rare type Ia supernovae explosions or electron-capture supernovae^31,75^.

### Calculation of mass-independent values of SiC grains

The average mass-independent μ^30^Si, ε^94,95^Mo and ε^96^Zr isotope compositions of presolar SiC grains are calculated using data from the presolar grain database^19^ and ε^145^Nd compositions were calculated using data from ref. ^76^. First, the raw isotope ratios for mainstream, X, Y and Z SiC grains are calculated from their mass-dependent values. Mass-dependent effects are then corrected for using true ^29^Si/^28^Si = 0.05080, ^98^Mo/^96^Mo = 1.45711, ^90^Zr/^94^Zr = 2.96030 and ^146^Nd/^144^Nd = 0.7219 ratios for internal normalization, assuming an exponential law. The μ^30^Si, ε^94,95^Mo, ε^96^Zr and ε^145^Nd compositions of each SiC grain is then the deviation of the mass-fractionation corrected ratios from the solar ratios. The average mass-independent values of all SiC grains combined is then calculated by assigning weights of 90% to mainstream grains, 2% to X grains, and 4% to both Y and Z grains, based on their relative abundances in meteorites^77^. Using this method, we derive the average SiC compositions reported in Extended Data Table 3. Given the anomalous Si isotopic compositions of SiC grains (μ^30^Si ≈ −2,700), we explore whether their heterogenous distribution in the disk could explain the observed μ^30^Si variability. To generate the most depleted μ^30^Si compositions as recorded by pyroxene pallasites (μ^30^Si = −11.0) from a solar composition approximated by CI chondrites (11 wt% Si, μ^30^Si = 32.8), requires a SiC concentration (70 wt% Si, μ^30^Si = −2,700) of 2,550 ppm, which exceeds meteoritic abundances by a factor of about 75.

### Pebble accretion numerical simulations

We use numerical simulations to explore whether the μ^30^Si composition of Mars and proto-Earth can be reproduced if their growth occurred by a combination of collisions and pebble accretion during the disk lifetime. Our terrestrial planet formation simulations are built on a model that has been described earlier^11,78,79^. The simulations consider the growth of a single planetary body within a protoplanetary disk. The protoplanetary disk is modelled as a standard, time-dependent alpha disk, where the mass accretion rate onto the star drops from an initial 10^−6^ *M*_☉_ yr^−1^ to 10^−9^ *M*_☉_ yr^−1^ over 5 Myr of evolution. The viscosity coefficient is set to *α* = 10^−2^. The planets grow by accreting pebbles and planetesimals. Pebble sizes are fixed at 1 mm, in agreement with observational constraints from protoplanetary disks^80^ as well as dominant sizes of chondrules found in chondritic meteorites. The diffusion coefficient of the gas sets the scale height of the pebbles and hence the transition from the initial three-dimensional accretion to more efficient two-dimensional pebble accretion. The diffusion coefficient is taken as *δ* = 3 × 10^−4^, which is significantly lower than the global *α* viscosity. This reflects that the mid-plane of the protoplanetary disk probably has only weak coupling between gas and magnetic fields, lowering the strength of the turbulence there. The radial pebble flux is calculated as a fraction *ξ* times the instantaneous flux of gas through the protoplanetary disk (with *ξ* decreasing with time owing to pebble drift; see caption of Extended Data Fig. 2 for details). We include a planetesimal belt centred around 1.6 au; we showed in ref. ^11^ that such a planetesimal belt yields a good match to the architecture of the terrestrial planets. The planets accrete a large fraction of planetesimals while inside this belt, but pebble accretion dominates for planetary masses above 0.1 *M*_E_ that migrate out of the belt. In this picture, Mars is a planetary embryo that did not migrate out of its birth region, whereas Venus, Earth and Theia experienced significant inwards migration^11^.

In these models, a first generation of planetesimals forms by the streaming instability followed by a mixture of collisional accretion of these planetesimals and pebble accretion to grow Mars-sized bodies. A transition to a growth mode dominated by pebble accretion occurs from this point, which is a natural consequence of increased gravitational focusing of pebbles onto a protoplanet relative to planetesimals when Mars mass is achieved^11^. We use the μ^30^Si composition defined by non-carbonaceous achondrite parent bodies as the starting composition of the first-generation planetesimals and fit a power law through the data of Fig. 1b for the Si composition as a function of time. The fit follows *t* = *C*(μ^30^Si − μ^30^Si_0_)^*β*^, where μ^30^Si_0_ is the value of μ^30^Si at *t* = 0, *β* is the power-law parameter and *C* is a fitted constant. The constants *C* and μ^30^Si_0_ are fully determined by requiring the function to fit the composition of the achondrites and the chondrites at their respective accretion times. In Extended Data Fig. 2, we present results for *β* = 1 (linear fit) and *β* = 0.5 (representing a more abrupt transition in the composition of the inner disk). The linear model yields an accretion age of 2 Myr, whereas the abrupter admixing of pristine dust to the inner Solar System yields an accretion age of approximately 3 Myr. The final mass of Earth includes a contribution from an additional terrestrial planet, Theia, that collided with the proto-Earth to form the Earth–Moon system at a later time. This early termination of Mars’ growth is a natural consequence of stirring of its orbit by other protoplanets in the competition for pebbles. Critically, the inferred formation timescales of the terrestrial planets cannot be extended by any reasonable parameter adjustments.

### Thermal processing in the planetary envelope

**Extended Data Fig. 9:**
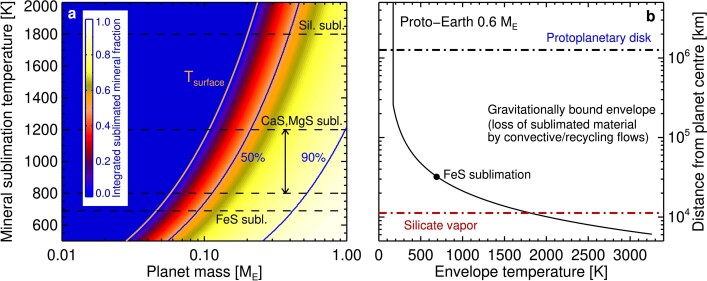
A) Proportion of accreted material that has been processed at a continuous range of mineral sublimation temperatures, as a function of planetary mass. We assumed here a planetary growth time-scale of 0.7 million years when constructing the temperature and pressure structure of the planetary envelope. The surface temperature of the planet is shown in orange. We mark thermal processing fractions of 50% and 90% with solid black lines. The sublimation temperature of troilite FeS (690 K), the range of sublimation temperatures of Cas and MgS (800 K −1,200 K) and the sublimation temperature of silicates (1,800 K) are marked with dotted lines. Significant thermal processing begins at a planetary mass of approximately 0.04 Earth masses. B) Thermal structure of the envelope of proto-Earth, assumed here to have a mass of 0.6 M_E_, shown with distance from the planetary centre along the y-axis. Silicates sublimate near the planetary surface and form a layer rich in SiO vapour. FeS sublimates in the gravitationally bound envelope; here the released sulfur (H_2_S) and embedded atoms such as Mo easily diffuse back to the protoplanetary disk via convective flows and recycling flows that penetrate from the disk into the envelope.

The gravity of the protoplanet attracts a hydrothermal envelope of hydrogen and helium that connects continuously to the pressure and temperature conditions in the protoplanetary disk at the Hill radius^39,81^. The sublimation of minerals within the accreted pebbles is calculated using an equilibrium model for the planetary envelope structure (that is, pressure and temperature as a function of height over the surface). We set the luminosity of the planet as *L* = *GM*_pla_${\dot{M} }_{{\rm{p}}{\rm{l}}{\rm{a}}}$/*R*_pla_ where *G* is the gravitational constant, *M*_pla_ is the planet’s mass, *R*_pla_ is the planet’s radius, and ${\dot{M} }_{{\rm{p}}{\rm{l}}{\rm{a}}}$ is the mass accretion rate set as exponential mass growth on timescale *Τ* = 0.7 Myr (following ref. ^41^). Here *R*_pla_ marks the surface of the protoplanet, which is initially solid but later melts from the accretion energy to form a magma ocean. We use analytical structure expressions that assume hydrostatic equilibrium and the minimum of the radiative and convective temperature gradient^81^. These expressions utilize a realistic power-law dependence of the opacity on the temperature (we set the opacity level at the disk temperature to 0.01 m^2^ kg^−1^). Extended Data Fig. 9a shows the proportion of the accreted material that has been processed at a continuous range of possible mineral sublimation temperatures as a function of planetary mass. Significant thermal processing begins at around 0.04 *M*_E_. For a Mars-mass planet, approximately 50% of the material has been processed at the FeS sublimation temperature of 690 K, while the processed fraction rises to >90% for a protoplanet of 0.6 *M*_E_. Extended Data Fig. 9b shows the structure of the envelope of proto-Earth, assuming a mass of 0.6 *M*_E_.

The Bondi radius defined as *R*_B_ = *GM*/*c*_s_^2^ demarks the extent of the gravitationally bound envelope, where *c*_s_ is the sound speed of the gas. For our proto-Earth model, *R*_B_ ≈ 5 × 10^5^ km. The regions of the Hill radius outside the Bondi radius are penetrated by recycling flows from the protoplanetary disk^39^. We assume that the H_2_S molecules that are released upon FeS sublimation, as well as a fraction of about 10% of the embedded trace elements (such as Mo and Ru), escape from the Bondi radius with large-scale upwards-moving convective cells^41^. Such low-abundance trace elements, once released from the FeS, will either remain in atomic form or nucleate as tiny nanoscale clusters that move easily with the gas flow. These clusters will not be able to grow to pebble sizes owing to the scarcity of the elements in the gas phase^82^. We can not a priori predict the mass loss of refractory elements released by FeS sublimation in the envelope. Our choice of loss fraction is motivated by the simultaneous match to the Mo composition of both Earth and Mars for a range of loss fractions between 7% and 10% (Fig. 3).

The gas flows in the Bondi radius have a characteristic length scale of *R*_B_ and a characteristic speed of *c*_s_ and hence carry a turbulent diffusion coefficient of *D* ≈ *α*_conv_*R*_B_*c*_s_, where *α*_conv_ is a factor of order unity in the convective envelope^41^. We estimate the timescale to diffuse over the Bondi radius as *t*_diff_ = *R*_B_^2^/(*α*_conv_*c*_s_*R*_B_) = *GM*/(*α*_conv_*c*_s_^3^) = 0.046 yr (*M*/*M*_E_) (*T*/125 K)^−3/2^*α*_conv_. This is clearly a very rapid timescale in the convective region where *α*_conv_ is approximately unity for the high-pebble-accretion luminosities^83^. For the radiative zone, even tiny amounts of turbulence (*α*_conv_ > 10^−7^) arising from of convective overshoot^83^ or stirring from the protoplanetary disk turbulence facilitates loss on a timescale of a million years. We note that this escape mechanism would not apply to more refractory minerals such as silicates, as silicate vaporization reduces the dust opacity and leads to formation an additional radiative region at a temperature of approximately 1,800 K that would prevent more refractory species from escaping via convective cells^41^. The sublimation of silicates also forms a layer rich in SiO vapour, with a mean molecular weight that is much higher than the hydrogen and helium gas in the envelope^84^. This mean-molecular-weight gradient further prevents convection from penetrating over the boundary between envelope and SiO layer. SiC grains will also sublimate in this silicate layer and hence add their isotopic contents to the bulk planet.

### Iron–nickel isotope evolution simulations

Each of the 100 Monte Carlo simulations plotted in Fig. 4 traces the evolution of the terrestrial core and mantle during two steps of Earth formation, namely accretion of outer Solar System pebbles and the Moon-forming giant impact. Group IIIAB iron meteorites and CI chondrites are used as isotopic proxies for inner and outer Solar System planetesimals and pebbles (starting composition in each simulation), respectively.

Ureilite meteorites have been taken as a proxy for the inner-disk composition in earlier studies^4^. However, limited Ni isotope data exist for ureilites and these are plagued by large uncertainties^85^. Thus, we use group IIIAB iron meteorites as a proxy for the composition of the disk material at early times. This is a reasonable assumption as group IIIAB iron meteorites are modelled to have accreted in the inner disk within the first million years of Solar System evolution^86^. Fe and Ni core–mantle partitioning was modelled with a constant core mass fraction of 0.325 (ref. ^87^) and at a fixed oxidation state corresponding to 6.26% of Fe in the mantle^50^. Ni partitioning was modelled to be pressure dependent, becoming significantly more siderophile under low pressure^50,88^. At the start of the simulation during pebble accretion, full equilibration between accreted metal component and the mantle before core segregation was assumed for each calculation step. During the giant impact, full equilibration between the proto-Earth and Theia mantles was assumed but different degrees of equilibration with Theia’s core were modelled as fractions of Theia core that equilibrated with the mantle. It is noted that the proto-Earth core remains isotopically isolated. An equilibration depth of 0.5 of the full mantle depth was chosen to achieve a final mantle equilibration pressure of about 55 GPa in accordance with previous work^89^. This results in a final *D*_Ni(metal/silicate)_ of about 26 (ref. ^88^). We assume masses of proto-Earth and Theia of 0.6 *M*_E_ and 0.4 *M*_E_ at the time of impact, following numerical simulations of pebble accretion produced by ref. ^11^. Both protoplanets are assumed to have the same bulk composition and accreted 26% of their masses from the CI reservoir, based on the Si isotope data from our study. For the Monte Carlo simulations, the compositions of group IIIAB iron meteorites and CI chondrites were varied according to a normal distribution with an s.d. equal to the s.e. of the data. Other input parameters were assumed to be invariant. All input parameters for these models are described in Extended Data Table 3.

We note that the Ni isotope composition of Earth’s mantle is not on a mixing relationship between achondrites (that is, group IIIAB iron meteorites) and CI chondrites on a μ^60^Ni–μ^62^Ni diagram. In detail, the terrestrial mantle plots above the mixing line between IIIAB and CI chondrites. Therefore, we explore whether the terrestrial ^60^Ni excess can be explained by the radioactive decay of the short-lived ^60^Fe nuclide (*t*_1/2_ ≈ 2.6 Myr). From the model provided in Fig. 4, we calculate that the Fe/Ni ratios of the mantles of proto-Earth and Theia after accretion and differentiation are about 163 and about 273, respectively. Accepting a mass of 0.6 *M*_E_ and 0.4 *M*_E_ for proto-Earth and Theia, respectively^11^, mass-balance arguments require that the combined mantles of proto-Earth and Theia have an Fe/Ni ratio of about 193 before equilibration with Theia’s core. We assume that the main differentiation of proto-Earth and Theia occurred at 2.5 Myr after Solar System formation, which represents the average of the two pebble accretion simulations presented in Extended Data Fig. 2. Using an initial Solar System ^60^Fe/^56^Fe abundance of 1.15 ± 0.26 × 10^−8^ (ref. ^85^), we calculate an excess μ^60^Ni of 3.6 ppm for the composition of the combined mantles of proto-Earth and Theia relative to the CI composition (representing the composition of the combined mantles before decay of ^60^Fe). We show in Extended Data Fig. 4d that once the contribution from radiogenic ^60^Ni is accounted for, the Ni isotope composition of the combined mantles of Earth and Theia before partial equilibration with Theia’s core aligns on a mixing relationship with Earth’s modern mantle and group IIIAB iron meteorites. It is noted that the degree of equilibration with Theia’s core required to account for the terrestrial composition is <20%, consistent with the Fe–Ni evolution model shown in Fig. 4. The presence of radiogenic ^60^Ni in Earth’s mantle is not only consistent with but also requires rapid formation of the proto-Earth by pebble accretion during the lifetime of ^60^Fe. The Ni isotope compositions of CI represent the average (±2 s.e.) value from refs. ^85,90,91^, whereas the IIIAB composition is from refs. ^61,85^. The terrestrial mantle composition is an average of two mean values calculated from refs. ^61,91^.

## Online content

Any methods, additional references, Nature Portfolio reporting summaries, source data, extended data, supplementary information, acknowledgements, peer review information; details of author contributions and competing interests; and statements of data and code availability are available at 10.1038/s41586-023-06135-z.

### Supplementary information

Supplementary Table 1 Mass-independent μ^30^Si and μ^29^Si values as well as mass-dependent δ^30^Si and δ^29^Si data relative to NBS-28.

Supplementary Table 2 Mass-independent μ^43^Ca and μ^48^Ca data.

### Source data

Source Data Fig. 1

Source Data Fig. 2

## Figures and Tables

**Extended Data Table 1:**
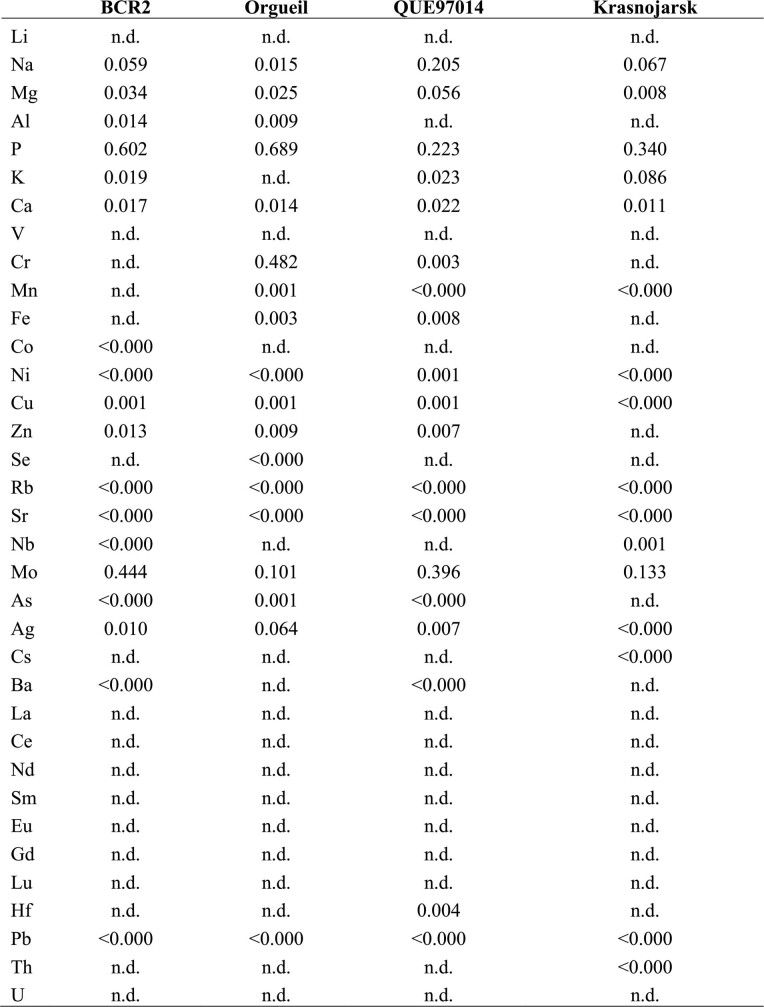
Residual impurities in purified Si fractions for various meteorite samples and BCR-2

Reported as % of the 4 ppm Si content of the solutions. n.d. not detected.

**Extended Data Table 2:**
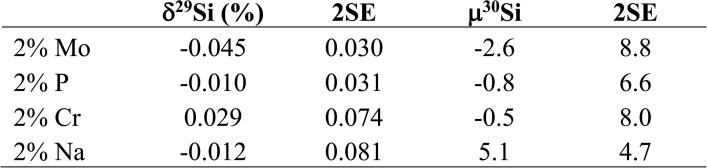
δ^29^Si and μ^30^Si values of the NBS-28 standard solution doped with various contaminants

The 2% doping level is approximately 3–10 times higher than that observed in our samples.

**Extended Data Table 3:**
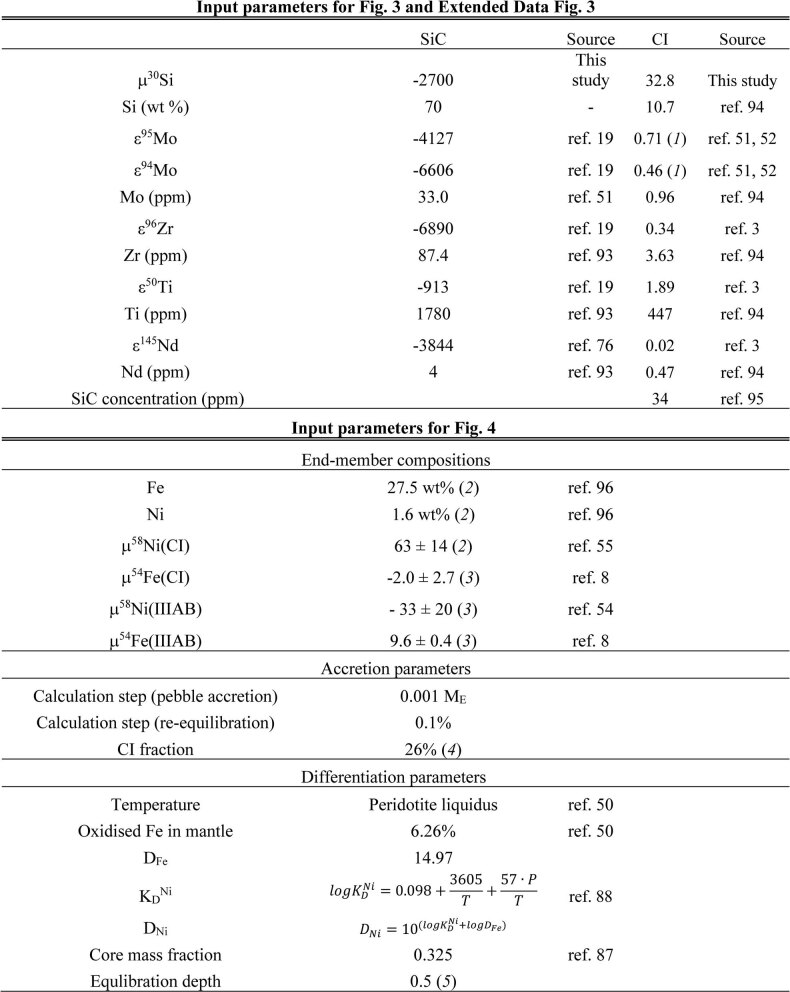
Input parameters for modelling of envelope processing and the iron-nickel isotope evolution of Earth

(1) Average of values from ref. ^51^ and ref. ^52^. (2) Identical for all accreting solids and bulk Proto-Earth, Theia and Earth. (3) Was modelled as a free parameter in Monte-Carlo simulations according to a normal distribution with standard deviation equal to the standard error of the data. (4) Identical for Proto-Earth and Theia. (5) As a fraction of the total mantle depth. Additional model parameters in the table not previously cited are from^93–96^.

## Data Availability

All data are available at EarthChem^92^. Source data are provided with this paper.

## References

[1] Alexander CMO’D (2022). An exploration of whether Earth can be built from chondritic components, not bulk chondrites. Geochim. Cosmochim. Acta.

[2] Trinquier A (2009). Origin of nucleosynthetic isotope heterogeneity in the solar protoplanetary disk. Science.

[3] Burkhardt C (2021). Terrestrial planet formation from lost inner Solar System material. Sci. Adv..

[4] Schiller M, Bizzarro M, Fernandes VA (2018). Isotopic evolution of the protoplanetary disk and the building blocks of Earth and the Moon. Nature.

[5] Dauphas N, Schauble EA (2016). Mass fractionation laws, mass-independent effects, and isotopic anomalies. Annu. Rev. Earth Planet. Sci..

[6] Chambers JE (2004). Planetary accretion in the inner Solar System. Earth Planet. Sci. Lett..

[7] van der Marel N, Dong R, di Francesco J, Williams JP, Tobin J (2019). Protoplanetary disk rings and gaps across ages and luminosities. Astrophys. J..

[8] Schiller M, Bizzarro M, Siebert J (2020). Iron isotope evidence for very rapid accretion and differentiation of the proto-Earth. Sci. Adv..

[9] Yu G, Jacobsen SB (2011). Fast accretion of the Earth with a late Moon-forming giant impact. Proc. Natl Acad. Sci. USA.

[10] Lambrechts M, Johansen A (2012). Rapid growth of gas-giant cores by pebble accretion. Astron. Astrophys..

[11] Johansen A (2021). A pebble accretion model for the formation of the terrestrial planets in the Solar System. Sci. Adv..

[12] Timmes FX, Clayton DD (1996). Galactic evolution of silicon isotopes: application to presolar SiC grains from meteorites. Astrophys. J..

[13] Clayton R, Hinton R, Davis A (1988). Isotopic variations in the rock-forming elements in meteorites. Phil. Trans. R. Soc. Lond. A.

[14] Javoy M, Balan E, Méheut M, Blanchard M, Lazzeri M (2012). First-principles investigation of equilibrium isotopic fractionation of O- and Si-isotopes between refractory solids and gases in the solar nebula. Earth Planet. Sci. Lett..

[15] Shahar A (2009). Experimentally determined Si isotope fractionation between silicate and Fe metal and implications for Earth’s core formation. Earth Planet. Sci. Lett..

[16] Zambardi T (2013). Silicon isotope variations in the inner solar system: implications for planetary formation, differentiation and composition. Geochim. Cosmochim. Acta.

[17] Rai VK, Murty SVS, Ott U (2003). Noble gases in ureilites: cosmogenic, radiogenic, and trapped components. Geochim. Cosmochim. Acta.

[18] Herzog GF (2015). Cosmic-ray exposure ages of pallasites. Meteorit. Planet. Sci..

[19] Stephan T (2020). The presolar grain database reloaded—silicon carbide. Lunar Planet. Sci..

[20] Huss GR, Meshik AP, Smith JB, Hohenberg CM (2003). Presolar diamond, silicon carbide, and graphite in carbonaceous chondrites: implications for thermal processing in the solar nebula. Geochim. Cosmochim. Acta.

[21] Bischoff A, Vogel N, Roszjar J (2011). The Rumuruti chondrite group. Chem. Erde.

[22] Alexander CMOD (2019). Quantitative models for the elemental and isotopic fractionations in the chondrites: the non-carbonaceous chondrites. Geochim. Cosmochim. Acta.

[23] Kruijer T, Burkhardt C, Budde G, Kleine T (2017). Age of Jupiter inferred from the distinct genetics and formation times of meteorites. Proc. Natl Acad. Sci. USA.

[24] Desch SJ, Kalyaan A, Alexander CMO (2018). The effect of Jupiter’s formation on the distribution of refractory elements and inclusions in meteorites. Astrophys. J. Suppl. Ser..

[25] Schiller M, Connelly JN, Glad AC, Mikouchi T, Bizzarro M (2015). Early accretion of protoplanets inferred from a reduced inner Solar System ^26^Al inventory. Earth Planet. Sci. Lett..

[26] Hevey PJ, Sanders IS (2006). A model for planetesimal meltdown by ^26^Al and its implications for meteorite parent bodies. Meteorit. Planet. Sci..

[27] Sugiura N, Fujiya W (2014). Correlated accretion ages and ε^54^Cr of meteorite parent bodies and the evolution of the solar nebula. Meteorit. Planet. Sci..

[28] Gradie J, Tedesco E (1982). Compositional structure of the Asteroid Belt. Science.

[29] Lichtenberg T, Drazkowska J, Schönbachler M, Golabek GJ, Hands TO (2021). Bifurcation of planetary building blocks during Solar System formation. Science.

[30] Liu B, Johansen A, Lambrechts M, Bizzarro M, Haugbølle T (2022). Natural separation of two primordial planetary reservoirs in an expanding solar protoplanetary disk. Sci. Adv..

[31] Clayton, D. *Handbook of Isotopes in the Cosmos* 140–150 (Cambridge Univ. Press, 2003).

[32] Ek M (2020). The origin of s-process isotope heterogeneity in the solar protoplanetary disk. Nat. Astron..

[33] Dauphas N, Pourmand A (2011). Hf–W–Th evidence for rapid growth of Mars and its status as a planetary embryo. Nature.

[34] Sanloup C, Jambon A, Gillet P (1999). A simple chondritic model of Mars. Phys. Earth Planet. Inter..

[35] Liebske C, Khan A (2019). On the principal building blocks of Mars and Earth. Icarus.

[36] Marty B (2012). The origins and concentrations of water, carbon, nitrogen and noble gases on Earth. Earth Planet. Sci. Lett..

[37] Grewal DS, Dasgupta R, Sun C, Tsuno K, Costin G (2019). Delivery of carbon, nitrogen, and sulfur to the silicate Earth by a giant impact. Sci. Adv..

[38] Schlichting HE, Sari R, Yalinewich A (2015). Atmospheric mass loss during planet formation: the importance of planetesimal impacts. Icarus.

[39] Lambrechts M, Lega E (2017). Reduced gas accretion on super-Earths and ice giants. Astron. Astrophys..

[40] Kurokawa H, Tanigawa T (2018). Suppression of atmospheric recycling of planets embedded in a protoplanetary disc by buoyancy barrier. Mon. Not. R. Astron. Soc..

[41] Johansen A, Nordlund Å (2020). Transport, destruction, and growth of pebbles in the gas envelope of a protoplanet. Astrophys. J..

[42] Mezger, K., Schönbächler, M. & Bouvier, A. Accretion of the Earth—missing components? *Space Sci. Rev.* **216**, 27 (2020).

[43] Imamura K, Honda M (1976). Distribution of tungsten and molybdenum between metal, silicate, and sulphide phases of meteorites. Geochim. Cosmochim. Acta.

[44] Hammouda, T., Boyet, M., Frossard, P. & Cartier, C. The message of oldhamites from enstatite chondrites. *Prog. Earth Planet. Sci.* **9**, 13 (2022).

[45] Yoshizaki T, Ash R, Lipella M, Yokoyama T, McDonough W (2021). Variable refractory lithophile element compositions of planetary building blocks: Insights from components of enstatite chondrites. Geochim. Cosmochim. Acta.

[46] Kama M (2019). Abundant refractory sulfur in protoplanetary disks. Astrophys. J..

[47] Dauphas N (2010). Neutron-rich chromium isotope anomalies in supernova nanoparticles. Astrophys. J..

[48] Burkhardt C, Dauphas N, Hans U, Bourdon B, Kleine T (2019). Elemental and isotopic variability in solar system materials by mixing and processing of primordial disk reservoirs. Geochim. Cosmochim. Acta.

[49] Drązkowska J, Alibert Y (2017). Planetesimal formation starts at the snow line. Astron. Astrophys..

[50] Wood B, Wade J, Kilburn M (2008). Core formation and the oxidation state of the Earth: additional constraints from Nb, V and Cr partitioning. Geochim. Cosmochim. Acta.

[51] Dauphas N, Marty B, Reisberg L (2002). Molybdenum nucleosynthetic dichotomy revealed in primitive meteorites. Astrophys. J..

[52] Burkhardt C (2011). Molybdenum isotope anomalies in meteorites: constraints on solar nebula evolution and origin of the Earth. Earth Planet. Sci. Lett..

[53] Render J, Brennecka G, Wang S, Wasylenki L, Kleine T (2018). A distinct nucleosynthetic heritage for early Solar System solids recorded by Ni isotope signatures. Astrophys. J..

[54] Nanne J, Nimmo F, Cuzzi J, Kleine T (2019). Origin of the non-carbonaceous–carbonaceous meteorite dichotomy. Earth Planet. Sci. Lett..

[55] Hopp T, Dauphas N, Spitzer F, Burkhardt C, Kleine T (2022). Earth’s accretion inferred from iron isotopic anomalies of supernova nuclear statistical equilibrium origin. Earth Planet. Sci. Lett..

[56] Georg RB, Reynolds BC, Frank M, Halliday AN (2006). New sample preparation techniques for the determination of Si isotopic compositions using MC-ICPMS. Chem. Geol..

[57] Delstanche S (2009). Silicon isotopic fractionation during adsorption of aqueous monosilicic acid onto iron oxide. Geochim. Cosmochim. Acta.

[58] Alexander, B. G. B., Heston, W. M. & Iler, R. K. The solubility of amorphous silica in water. *J. Phys. Chem.* **58**, 453–455 (1954).

[59] Chen X, Lapen TJ, Chafetz HS (2017). Accurate and precise silicon isotope analysis of sulfur- and iron-rich samples by MC-ICP-MS. Geostand. Geoanal. Res..

[60] Paton C, Hellstrom J, Paul B, Woodhead J, Hergt J (2011). Iolite: freeware for the visualisation and processing of mass spectrometric data. J. Anal. At. Spectrom..

[61] Steele RCJ, Elliott T, Coath CD, Regolus M (2011). Confirmation of mass-independent Ni isotopic variability in iron meteorites. Geochim. Cosmochim. Acta.

[62] Pringle, E. A., Savage, P. S., Jackson, M. G., Barrat, J. A. & Moynier, F. Si isotope homogeneity of the solar nebula. *Astrophys. J.* **779**, 123 (2013).

[63] Dauphas N, Poitrasson F, Burkhardt C, Kobayashi H, Kurosawa K (2015). Planetary and meteoritic Mg/Si and δ^30^Si variations inherited from solar nebula chemistry. Earth Planet. Sci. Lett..

[64] Armytage RMG, Georg RB, Savage PS, Williams HM, Halliday AN (2011). Silicon isotopes in meteorites and planetary core formation. Geochim. Cosmochim. Acta.

[65] Chakrabarti R, Jacobsen SB (2010). Silicon isotopes in the inner Solar System: implications for core formation, solar nebular processes and partial melting. Geochim. Cosmochim. Acta.

[66] Fitoussi C, Bourdon B, Kleine T, Oberli F, Reynolds BC (2009). Si isotope systematics of meteorites and terrestrial peridotites: implications for Mg/Si fractionation in the solar nebula and for Si in the Earth’s core. Earth Planet. Sci. Lett..

[67] Pringle EA, Moynier F, Savage PS, Badro J, Barrat JA (2014). Silicon isotopes in angrites and volatile loss in planetesimals. Proc. Natl Acad. Sci. USA.

[68] Pringle EA, Savage PS, Badro J, Barrat JA, Moynier F (2013). Redox state during core formation on asteroid 4-Vesta. Earth Planet. Sci. Lett..

[69] Savage PS, Moynier F (2013). Silicon isotopic variation in enstatite meteorites: clues to their origin and Earth-forming material. Earth Planet. Sci. Lett..

[70] Sikdar J, Rai VK (2017). Simultaneous chromatographic purification of Si and Mg for isotopic analyses using MC-ICPMS. J. Anal. At. Spectrom..

[71] Zambardi T, Poitrasson F (2011). Precise determination of silicon isotopes in silicate rock reference materials by MC-ICP-MS. Geostand. Geoanalytical Res..

[72] Schiller M, Paton C, Bizzarro M (2012). Calcium isotope measurement by combined HR MC-ICPMS and TIMS. J. Anal. At. Spectrom..

[73] Schiller M, Paton C, Bizzarro M (2015). Evidence for nucleosynthetic enrichment of the protosolar molecular cloud core by multiple supernova events. Geochim. Cosmochim. Acta.

[74] Hoppe P, Leitner J, Kodolány J (2021). Isotope systematics of presolar silicate grains: new insights from magnesium and silicon. Astrophys. J..

[75] Wanajo S, Janka H-T, Müller B (2013). Electron-capture supernovae as origin of ^48^Ca. Astrophys. J. Lett..

[76] Yin QZ, Lee CTA, Ott U (2006). Signatures of the s‐process in presolar silicon carbide grains: barium through hafnium. Atrophys. J..

[77] Zinner, E. in *Treatise on Geochemistry Update* (eds Holland, H. D. et al.) 1–33 (Elsevier, 2007).

[78] Johansen A, Bitsch B (2019). Exploring the conditions for forming cold gas giants through planetesimal accretion. Astron. Astrophys..

[79] Johansen A, Ida S, Brasser R (2019). How planetary growth outperforms migration. Astron. Astrophys..

[80] Carrasco-González C (2019). The radial distribution of dust particles in the HL Tau disk from ALMA and VLA observations. Atrophys. J.

[81] Piso A, Youdin A (2014). On the minimum core mass for giant planet formation at wide separations. Atrophys. J..

[82] Johansen, A. & Dorn, C. Nucleation and growth of iron pebbles explains the formation of iron-rich planets akin to Mercury. *Astron. Astrophys.* **662**, A19 (2022).

[83] Popovas, A., Nordlund, Å. & Ramsey, J. P. Pebble dynamics and accretion on to rocky planets—II. Radiative models. *Mon. Not. R. Astron. Soc.* **482**, L107–L111 (2018).

[84] Brouwers, M. G. & Ormel, C. W. How planets grow by pebble accretion-II. Analytical calculations on the evolution of polluted envelopes. *Astron. Astrophys.* **634**, A15 (2020).

[85] Tang H, Dauphas N (2012). Abundance, distribution, and origin of ^60^Fe in the solar protoplanetary disk. Earth Planet. Sci. Lett..

[86] Spitzer F, Burkhardt C, Nimmo F, Kleine T (2021). Nucleosynthetic Pt isotope anomalies and the Hf–W chronology of core formation in inner and outer Solar System planetesimals. Earth Planet. Sci. Lett..

[87] Wang, H. S., Lineweaver, C. H. & Ireland, T. R. The elemental abundances (with uncertainties) of the most Earth-like planet. *Icarus* **299**, 460–474 (2018).

[88] Siebert J, Badro J, Antonangeli D, Ryerson F (2012). Metal–silicate partitioning of Ni and Co in a deep magma ocean. Earth Planet. Sci. Lett..

[89] Badro J (2015). Core formation and core composition from coupled geochemical and geophysical constraints. Proc. Natl Acad. Sci. USA.

[90] Steele RC, Coath CD, Regelous M, Russell S, Elliott T (2012). Neutron-poor nickel isotope anomalies in meteorites. Atrophys. J..

[91] Makhatadze GV, Schiller M, Bizzarro M (2023). High precision nickel isotope measurements of early Solar System materials and the origin of nucleosynthetic disk variability. Geochim. Cosmochim. Acta.

[92] Onyett, I. et al. Silicon isotope data for bulk meteorites and terrestrial standards, version 1.0. *IEDA*10.26022/IEDA/112740 (2022).

[93] Amari S, Hoppe P, Zinner E, Lewis RS (1995). Trace-element concentrations in single circumstellar silicon carbide grains from the Murchison meteorite. Meteoritics.

[94] Palme, H., Lodders, K. & Jones, A. in *Treatise on Geochemistry* (ed. Davis, A. M.) 15–36 (Elsevier, 2014).

[95] Davidson J (2014). Abundances of presolar silicon carbide grains in primitive meteorites determined by NanoSIMS. Geochim. Cosmochim. Acta.

[96] Wasson JT, Kallemeyn GW (1988). Compositions of chondrites. Phil. Trans. R. Soc. Lond..

